# Arterial Stiffness, Subendocardial Impairment, and 30-Day Readmission in Heart Failure Older Patients

**DOI:** 10.3389/fcvm.2022.918601

**Published:** 2022-06-17

**Authors:** Francesco Fantin, Anna Giani, Arianna Franconi, Elena Zoico, Silvia Urbani, Andrea P. Rossi, Gloria Mazzali, Mauro Zamboni

**Affiliations:** ^1^Section of Geriatric Medicine, Department of Medicine, University of Verona, Verona, Italy; ^2^Section of Geriatric Medicine, Department of Surgery, Dentistry, Pediatric, and Gynecology, University of Verona, Verona, Italy

**Keywords:** heart failure, SEVR, PWV, hospital readmission, older patients

## Abstract

Arterial stiffness and subendocardial perfusion impairment may play a significant role in heart failure (HF) outcomes. The aim of the study was to examine the main predictors of 30-day readmission in geriatric patients, hospitalized with HF, explore hemodynamical parameters, arterial stiffness indexes, and subendocardial viability ratio (SEVR). In total, 41 hospitalized patients, affected by HF, were included; they underwent clinical evaluation, routine laboratory testing, and echocardiography. At the time of admission, after the achievement of clinical stability (defined as switching from intravenous to oral diuretic therapy), and at discharge, arterial tonometry was performed to evaluate carotid-femoral pulse wave velocity (PWVcf) and SEVR (then corrected for hemoglobin concentration and oxygen saturation). Through the evaluations, a significant progressive decrease in PWVcf was described (17.79 ± 4.49, 13.54 ± 4.54, and 9.94 ± 3.73 m/s), even after adjustment for age, gender, mean arterial pressure (MAP) variation, and left ventricular ejection fraction (LVEF). A significant improvement was registered for both SEVR (83.48 ± 24.43, 97.94 ± 26.84, and 113.29 ± 38.02) and corrected SEVR (12.74 ± 4.69, 15.71 ± 5.30, and 18.55 ± 6.66) values, and it was still significant when adjusted for age, gender, MAP variation, and LVEF. After discharge, 26.8% of patients were readmitted within 30 days. In a multivariate binary logistic regression analysis, PWVcf at discharge was the only predictor of 30-day readmission (odds ratio [OR] 1.957, 95% CI 1.112–3.443). In conclusion, medical therapy seems to improve arterial stiffness and subendocardial perfusion in geriatric patients hospitalized with heart failure. Furthermore, PWVcf is a valid predictor of 30-day readmission. Its feasibility in clinical practice may provide an instrument to detect patients with HF at high risk of rehospitalization.

## Introduction

Heart Failure (HF) is a frequent cardiovascular disorder, which shows a sharp increase in prevalence among the elderly (1, 2), with a remarkable clinical and comprehensive burden. Different patterns of HF have been described, and they account for different outcomes (3) and consequently different management of the acute and chronic phases of the disease. Therefore, detecting different subsets of HF is of paramount importance to tailor the treatment and the management of every patient and to provide effective results in terms of outcomes.

Arterial stiffness can be considered the morpho-physiologic expression of vascular aging, and it is strongly entailed in HF development, primarily affecting the diastolic function (4). Cardiovascular aging is characterized by a complex network of inflammatory and atherogenic pathways (5), which leads to reduced arterial wall elasticity. A progressive decrease of arterial compliance (6) results in increased blood pressure (BP), and mostly, in systolic blood pressure (SBP). Pressure overload drives cardiac remodeling, involving left ventricular (LV) wall thickening and left atrial dilation (4). Tonometric evaluation of pulse wave velocity (PWV), and in particular, carotid-femoral PWV (PWVcf) is a reliable predictor of mortality risk in different subsets of patients (7, 8), and it is a part of the latest European guidelines on arterial hypertension (9). Furthermore, interesting evidence points out a relationship between PWV and HF (10) showing worse outcomes in patients with HF with increased PWV (11, 12) and in HF with reduced ejection fraction (HFrEF) patients with reduced pulse pressure (PP) (10, 13).

Lately, several studies shed light on subendocardial perfusion impairment, which can be easily estimated by using subendocardial viability ratio (SEVR) provided by pulse wave analysis (PWA) (14), as the ratio between diastolic pressure time index (DPTI) and systolic pressure time index (SPTI) (15). Considering DTPI, which is the area under the diastolic phase in the aortic pulse wave profile, as an estimation of myocardial oxygen supply, STPI (the area under the systolic phase) as an index of cardiac tissue oxygen consumption (16), and SEVR represents myocardial workload, oxygen supply, and perfusion (17). Therefore, low values of SEVR mirror an impaired subendocardial perfusion (15). SEVR is related to several pathological conditions, such as chronic kidney disease (18), metabolic syndrome (19) hypertension (20), and orthostatic hypotension (21), peripheral arterial disease (22), and aortic stenosis (23), but still, less is known about the relationship between SEVR and HF in geriatric patients.

Although their roles have not been completely explored, hemodynamical parameters may represent a remarkable tool to stratify patients with HF during the in-hospital stay and early after discharge.

Furthermore, geriatric patients affected by HF often show an increased hospital admission or emergency department visits, due to worsening HF symptoms (24–26). Developing and intriguing research is looking for possible predictors of rehospitalization (24) and great importance is given to the “vulnerable time,” which immediately follows the discharge for acute HF (27).

Therefore, the aims of the present study were to evaluate arterial stiffness parameters in a cohort of consecutive in-patients affected by HF, to describe their trend over the time of in-hospital staying, and to identify whether any of these parameters may predict early readmission.

## Materials and Methods

### Study Population

The study cohort consisted of 41 consecutive patients (14 men and 27 women), aged over 70 years, hospitalized at the Geriatric Section of Verona University Hospital, affected by HF, and prospectively enrolled. The diagnosis of HF was made upon clinical presentation and suggestive chest X-ray imaging. All patients were prescribed intravenous diuretic medications. Patients with tachyarrhythmias with heart rate (HR) > 120 bpm (which would prevent the proper evaluation of arterial stiffness) and severe behavioral disorders (which would prevent a proper compliance during the evaluation) were excluded.

Each patient underwent a comprehensive clinical evaluation; demographical and clinical data included age, sex, BP, HR, and body weight measurement (Salus scale, Milan, Italy). A detailed clinical history was recorded, with particular interest in the presence of atrial fibrillation (AF), coronary artery disease (CAD), arterial hypertension, chronic kidney disease (defined as the presence of an estimated glomerular filtration rate (eGFR), by the Cockroft-Gault equation, lower than 60 ml/min/1.73 m^2^), and type 2 Diabetes Mellitus (DM2). Functional status was evaluated by Activity Daily Living (ADL) scale and Instrumental Activity Daily Living (IADL) scale. Cognitive profile was described by Mini-Mental State Examination (MMSE) and Montreal Cognitive Assessment (MOCA) test; whenever the MMSE score was > 24, the MOCA test was performed. A geriatric depression scale (GDS) was performed to investigate the psychological condition; Mini Nutritional Assessment (MNA) was used to evaluate the nutritional status. Charlson Comorbidity Index (CCI) was calculated for each patient, considering the Charlson age-adjusted index higher than 5 points as a predictor of poor clinical outcomes (28).

Brachial BP was measured twice using an aneroid sphygmomanometer (Heine Optothecnik, Gilching, Germany) in the subject’s non-dominant arm. The average of the readings was considered as the subject’s BP. Mean arterial pressure (MAP) was then derived, following the formula: MAP (mmHg) = DBP + 1/3 (SBP − DBP). PP, strongly related to cardiovascular risk and coronary heart disease (29, 30), was derived as the difference between SBP and DBP.

During the in-hospital stay, each patient underwent an echocardiographic evaluation, performed by experienced echocardiographers, and the left ventricular ejection fraction (LVEF) was therefore listed.

At the time of admission, venous blood samples for all metabolic assessments were obtained. Creatinine was measured by a modular analyzer (Roche Cobas 8000; Monza, Italy). eGFR was calculated by a Cockroft-Gault formula. Hemoglobin, albumin, and N-terminal pro-brain natriuretic peptide (nt-proBNP) were also measured.

### Arterial Stiffness and Subendocardial Viability Ratio Evaluation

The PWA was performed non-invasively using a small portable device called PulsePen (Diatecne, Milan, Italy) (31). Its software, WPulsePen 2.3.2, provides central aortic pressure values, an assessment of arterial pulse wave contours, estimation of reflection waves, and measurements of PWV. A detailed description of PWA calculation was provided in our previous studies (19, 21). PWA provides information about different arterial segments, as it can analyze both elastic arteries by the PWVcf and peripheral muscular arteries by carotid-radial PWV (PWVcr) (32).

PulsePen software also defines the augmentation pressure (AP), which is the systolic pressure increment caused by the reflection wave (from the periphery to the center), and the augmentation index (AI), which is the ratio between augmentation pressure and PP.

As previously described (19, 21), PulsePen software, by PWV traces analysis, provides SEVR measurement, which represents an indirect estimation of myocardial perfusion, relative to left ventricle workload. SEVR is derived as the ratio between DTPI (which is the area under the diastolic phase in the aortic profile, representing an estimation of myocardial oxygen supply) and STPI (the area under the systolic phase, representing cardiac tissue oxygen consumption) (16). Thus, SEVR indirectly reflects the adequacy of subendocardial perfusion.

A critical threshold for SEVR of 0.5 (50%) has been suggested (33), above which an insufficient subendocardial perfusion may be detected. As suggested by previous studies (16), since subendocardial oxygen supply depends both on coronary flow and arterial oxygen saturation, and it can be compromised in case of severe anemia or hypoxia, SEVR was adjusted for both hemoglobin and arterial oxygen concentration (34), following the formulas: SEVR CaO_2_ 1/4 CaO_2_ DTPI/STPI and CaO_2_ 1/41.34 × blood hemoglobin concentration (g/dl) × arterial oxygen saturation (%) + 0.003 × arterial pressure of oxygen (mmHg). According to previous evidence, the critical value for SEVR CaO_2_ was considered 10 (34).

Within 24 h from admission, after clinical stability achievement (which was considered as the switching time from intravenous to oral diuretics administration) and at discharge time, both PWV and SEVR were measured, by applanation tonometry. BP was noted each time (Heine Optothecnik, Gilching, Germany). MAP was then derived.

### Evaluation of 30-Day Readmission

One month after discharge, a telephonic follow-up checked the outcome as alive/dead and whether the patient had been readmitted to the hospital or not.

The study was approved by the Ethical Committee of the University of Verona.

### Statistical Analyses

Results are shown as mean value ± standard deviation (SD). Variables not normally distributed were log-transformed before analysis. Independent samples *t*-tests were used to compare baseline characteristics of patients with EF ≥ or < 50%. Analysis of variance (ANOVA) was performed when comparing continuous data throughout the follow-time, at admission, clinical stability, and discharge time. The analysis was then adjusted for age, gender, MAP variation, and LVEF.

Binary regression analysis was performed to evaluate 30-day readmission predictors, considering as independent variables age, gender, MAP, AF, PWVcf at discharge time, EF, eGFR, ADL, MMSE; DM2, previous HF hospitalization, CCI, albumin concentration, and length of in-hospital stay.

In order to evaluate the correlation between subclinical organ damage (PWV > 10 m/s) and re-hospitalization, a chi-square test was performed.

A significance threshold level of 0.05 was used throughout the study. All statistical analyses were performed using SPSS 23.0 version for Windows (IBM, Armonk, New York, NY, United States).

## Results

Our study population included 41 individuals, mean age 85.7 ± 5.5 years, of whom 27 were women. The main baseline characteristics of the study population are listed in Table 1. When considering the 30-day outcome, 26.8% (*n* = 11) of the overall population was re-hospitalized and 7.3% (*n* = 3) of patients died.

**TABLE 1:** Characteristics of the study population.

*n* = 41	Total (*n* = 41)	EF ≥ 50% (*n* = 26)	EF < 50% (*n* = 15)	*p*-value
Age (years)	85.68 ± 5.5	85.44 ± 5.35	86.00 ± 6.08	0.771
Hemoglobin (g/dl)	11.64 ± 1.84	11.72 ± 1.71	11.52 ± 5.52.11	0.776
Albumin (mg/dl)	36.12 ± 3.31	36.08 ± 3.58	36.18 ± 2.91	0.930
Creatinine (μmol/l)	112.85 ± 54.56	106.20 ± 49.38	123.93 ± 62.45	0.358
eGFR (ml/min)	45.48 ± 19.56	46.40 ± 19.99	43.93 ± 19.41	0.703
Nt-proBNP (pg/ml)	7659.40 ± 9502.59	3228.08 ± 2049.42	15044.93 ± 12281.71	<0.001
PWV cf (m/s)	17.79 ± 4.49	15.89 ± 5.22	16.39 ± 5.31	0.776
SEVR (%)	83.48 ± 24.43	83.00 ± 24.66	86.07 ± 29.62	0.739
Corrected SEVR	12.74 ± 4.69	12.70 ± 5.04	13.10 ± 5.41	0.820
AP (mmHg)	8.72 ± 4.52	10.01 ± 4.81	8.84 ± 4.65	0.454
AI	13.34 ± 5.49	15.26 ± 5.57	15.13 ± 5.91	0.944
PP (mmHg)	62.68 ± 12.37	64.16 ± 12.02	55.53 ± 9.18	0.015
SBP	132.74 ± 16.25	133.56 ± 14.09	125.53 ± 16.06	0.121
DBP	73.06 ± 8.33	72.80 ± 7.22	74.67 ± 9.34	0.513
MAP	96.35 ± 9.67	96.64 ± 8.10	94.67 ± 11.09	0.555
CAD [*n*,(%)]	10, (24.4)	4, (15.4)	6, (40)	0.13
CCI	7.28 ± 2.06	7.28 ± 2.20	7.27 ± 1.87	0.984
MMSE	23.50 ± 5.15	23.27 ± 6.54	23.55 ± 3.91	0.868
ADL	4.48 ± 1.88	4.84 ± 1.70	3.87 ± 2.06	0.136
IADL	4.10 ± 2.65	4.28 ± 2.63	3.80 ± 2.75	0.592
GDS	2.43 ± 1.94	2.32 ± 2.21	2.60 ± 1.45	0.632
In-hospital stay (days)	12.26 ± 4.85	12.84 ± 5.42	11.26 ± 1.94	0.307

*PWVcf, pulse wave velocity carotid-femoral; SEVR, sub-endocardial viability ratio; SBP, systolic blood pressure; DBP, diastolic blood pressure; MAP, mean arterial pressure; AP, augmentation pressure; AI, augmentation index; PP, pulse pressure; CAD, coronary artery disease; MMSE, mini mental state examination; ADL, activities daily living; IADL, instrumental activities daily living; GDS, geriatric depression scale.*

None of the baseline hemodynamic variables was different at baseline between patients readmitted after 30 days and patients not readmitted.

The cohort was then sorted in two different subgroups, relying on the echocardiographic EF: 26 (63%) subjects showed preserved EF (EF ≥ 50%), while 15 (37%) patients constituted a reduced EF subgroup, presenting EF < 50%.

Compared to preserved EF subjects, patients with reduced EF showed significantly higher concentrations of nt-proBNP (15,044.93 ± 1, 2281.71 vs. 3,228.08 ± 2,049.42 pg/ml, *p* < 0.001; Table 1).

Among the hemodynamic variables considered, PP resulted in the only one significantly difference between the two HF subgroups: at the time of admission, it was higher in individuals with preserved EF than in a reduced EF subgroup (64.16 ± 12.02 vs. 55.53 ± 9.18 mmHg, *p* = 0.015).

The comorbidity burden, evaluated by CCI and the duration of in-hospital stay, did not show any statistical difference between subpopulations.

**FIGURE 1:**
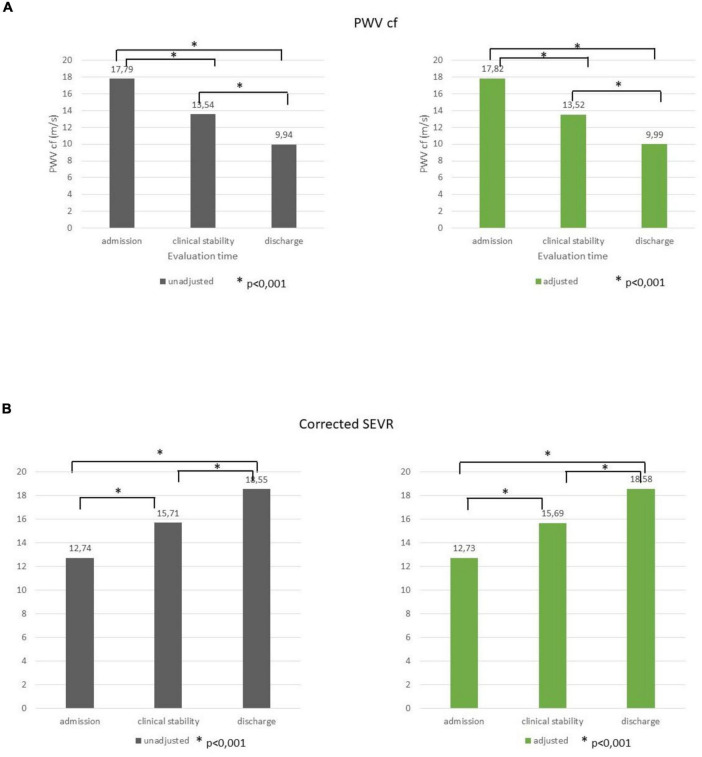
PWVcf **(A)** and corrected SEVR **(B)** values at admission, clinical stability, and at discharge before and after adjustment for age, gender, MAP variation, and LVEF. *p < 0.001.

Through the three evaluations, a progressive decrease in PWVcf was described (17.79 ± 4.49, 13.54 ± 4.54, and 9.94 ± 3.73 m/s, respectively, *p* < 0.001); the trend remained significant even after adjustment for age, gender, MAP variation, and LVEF (*p* < 0.001; Figure 1A).

Systolic BP, DBP, and MAP also displayed a decremental trend during the evaluations (Table 2, *p* < 0.001).

**TABLE 2:** Hemodynamic variables at admission, clinical stability, and at discharge.

*n* = 41	Admission (*t*1)	Clinical stability (*t*2)	Discharge (*t*3)	*p* for trend
PWV cf (m/s)	17.79 ± 4.49	13.54 ± 4.54	9.94 ± 3.73	<0.001
SEVR (%)	83.48 ± 24.43	97.94 ± 26.84	113.29 ± 38.02	<0.001
Corrected SEVR	12.74 ± 4.69	15.71 ± 5.30	18.55 ± 6.66	<0.001
AP (mmHg)	8.72 ± 4.52	5.73 ± 2..79	4.19 ± 1.89	<0.001
AI (%)	14.34 ± 5.49	10.91 ± 4.07	7.57 ± 2.95	<0.001
PP (mmHg)	62.68 ± 12.37	54.77 ± 10.93	46.51 ± 10.59	<0.001
SBP (mmHg)	132.74 ± 16.25	112.81 ± 14.86	120.23 ± 14.89	<0.001
DBP (mmHg)	73.06 ± 8.33	65.81 ± 6.59	62.58 ± 6.30	<0.001
MAP (mmHg)	96.35 ± 9.67	87.87 ± 9.22	82.97 ± 8.8	<0.001

*PWVcf, pulse wave velocity carotid-femoral; SEVR, sub-endocardial viability ratio; AP, augmentation pressure; AI, augmentation index; PP, pulse pressure; SBP, systolic blood pressure; DBP, diastolic blood pressure; MAP, mean arterial pressure.*

A significant improvement was registered for both SEVR (83.48 ± 24.43, 97.94 ± 26.84, and 113.29 ± 38.02, respectively) and corrected SEVR (12.74 ± 4.69, 15.71 ± 5.30, and 18.55 ± 6.66, respectively) values (*p* < 0.001 for both), and it was still significant when adjusted for age, gender, MAP variation, and LVEF (*p* < 0.001; Figure 1B).

In a multivariate binary logistic regression analysis (Table 3), considering 30-day readmission as a dependent variable and age, gender, EF, eGFR, ADL, MMSE, CCI, DM2, AF, previous hospitalizations due to HF, MAP at discharge, and PWVcf at discharge as independent variables. PWVcf at discharge was the only predictor of readmission (odds ratio [OR] 1.957, 95% CI1.112–3.443, *p* = 0.020).

**TABLE 3:** Binary logistic regression considering 30-day hospital readmission as a dependent variable and age, gender, EF, eGFR, ADL, DM2, AF, previous HF, and MAP at discharge as independent variables.

	β	S.E.	OR	C.I.	*p*-value
Age (years)	0.074	0.119	1.077	0.854–1.359	0.530
Male gender	0.262	1.576	1.300	0.059–28.531	0.868
EF (%)	–0.077	0.072	0.926	0.804–1.065	0.280
eGFR (ml/min)	0.030	0.035	1.031	0.962–1.105	0.391
Hemoglobin (g/dl)	–0.289	0.335	0.749	0.389–1.444	0.389
Albumin (mg/dl)	0.253	0.187	1.288	0.894–1.858	0.175
ADL	0.037	0.412	1.037	0.463–2.325	0.929
MMSE	–0.206	0.174	0.814	0.579–1.144	0.235
DM2	–1.096	1.397	0.334	0.022–5.170	0.433
AF	–0.796	1.430	0.451	0.027–7.437	0.578
Previous HF	0.921	1.208	2.511	0.235–26.810	0.446
MAP at discharge	–0.200	0.105	0.819	0.667–1.005	0.056
PWV cf at discharge	0.671	0.288	1.957	1.112–3.443	0.020
CCI	0.289	0.427	1.335	0.578–3.084	0.499
In-hospital stay (days)	0.114	0.149	1.120	0.837–1.500	0.446

*PWVcf at discharge, CCI, and length of hospitalization as an independent variable in the study population.*

*ADL, activities daily living; IADL, instrumental activities daily living; DM2, diabetes mellitus type 2; AF, atrial fibrillation; HF, heart failure; PWVcf, pulse wave velocity carotid-femoral; CCI, Charlson Comorbidity Index.*

Therefore, for each m/s of PWVcf increase at discharge time, we might assume a 95% increase in readmission risk.

Considering PWVcf = 10 m/s as threshold value for subclinical vascular damage (6) and excluding the deceased patients (*n* = 3), 8 over a total of 13 patients with PWVcf ≥ 10 m/s underwent rehospitalization within 30 days from discharge, whereas only 3 over a total of 25 individuals with PWVcf < 10 m/s were actually readmitted (*p* = 0.001).

## Discussion

Our study, led on HF patients, shows that PWVcf decreases with the optimization of the medical therapy and, irrespective of EF, SEVR increases in line with the clinical improvement. Moreover, the greater the PWV at discharge, the higher the risk of hospital readmission within the following 30 days. All the arterial stiffness parameters (PWVcf, PP, and AP e AI) and SEVR were improved along with the three evaluations during in-hospital stay, and this result was independent of the EF.

Carotid-femoral pulse wave velocity significantly decreases throughout the three evaluations, reflecting clinical improvement, even after adjustment for several variables (age, gender, MAP variation, and EF). These findings confirm and complement, since observed during hospitalization, previous evidence by Kim et al., who showed that PWV decreases in patients with HF after the achievement of clinical stability, which was evaluated after 3 months from discharge (35). These results are also in line with Zheng et al. (36) who found improvement in PWV in patients with hypertension after hypotensive treatment. We may speculate that in patients with HF, a proper medical therapy may improve cardiac pre-load and after-load resulting thus in reduced arterial wall stress and, probably, leading to a decrease in PWV.

To the best of our knowledge, this is the first study, which evaluated tonometric SEVR at admission, after the achievement of clinical stability, and at discharge time. Interestingly, SEVR and corrected-SEVR progressively increased throughout the three evaluations, supporting a relatively quick improvement in subendocardial perfusion in response to medical therapy.

Interestingly, as subendocardial oxygen delivery relies not only on coronary blood flow but also on arterial oxygen concentration, the observed improvement of corrected SEVR in our patients can more precisely represent the actual balance between arterial perfusion and oxygen delivery (16).

After the achievement of clinical stability, the improvement in ventricular workload as a consequence of the medical therapy, and, the reduced wall stress, may play a compelling role in increasing subendocardial perfusion. Corrected SEVR has been considered a feasible and reproducible technique, aimed at detecting preclinical organ damage in patients with HF (14); furthermore, as far as we could demonstrate with our data, corrected SEVR may be also useful to test eventual improvement after proper medical treatment.

Rehospitalization is a relevant problem in patients with HF. More than 1 patient over 4 were readmitted to the hospital in our study population after 30 days. Our readmission rate was more frequent than those observed in some previous studies (37–39) over the same follow-up time, yet the discrepancies could be explained by the age of the patients (much older in our population than in the others (37–39), high comorbidity burden (which was evaluated in our study, whether not entirely specified in other ones), and in line with Molvin et al. who observed a 33.5% readmission rate, in a Swedish cohort of 268 patients (mean age 75 years) over a follow-up time of 5 months (40).

Moreover, we could demonstrate that PWVcf, at discharge time, even after the adjustment for several independent variables (age, gender, EF, eGFR, ADL, MMSE, CCI, DM2, AF, previous HF hospitalization, and MAP at discharge), was the only significant predictor of rehospitalization. Subjects with PWVcf greater than 10 m/s, which is considered a marker of subclinical organ damage by the European Society of Hypertension Guidelines, showed more than twofold higher risk of readmission within the first 30 days after discharge when compared with those with values lower than 10 m/s (61% rehospitalization, as compared to the 26% recorded). Thus, our data show that PWVcf, besides being a valid marker of long-term subclinical organ damage both in the general population and in patients with increased cardiovascular risk (41–44), may be a predictor of early hospital readmission in patients with HF. These findings are in line and complement previous observations that PWV in patients with HFrEF, as evaluated by ultrasound, could predict death or hospitalization, over a follow-up time of 36 ± 19 months (45).

Limitations of our study should be acknowledged to interpret the results. First, the small sample size of our cohort may affect the statistical significance of some associations; moreover, we were neither allowed to sort the study population into the three HF subcategories suggested by ESC guidelines [HFrEF, HFmrEF, and HFpEF, (3)] nor to subdivide women and men populations. Furthermore, we did not know the precise cause of mortality or hospital readmission, which may have been of interest, in order to consider the burden of HF as compared to other comorbidities.

Nonetheless, the strength of our study was that we were able to evaluate PWVcf and SEVR for three consecutive times, providing a detailed trend of arterial stiffness and subendocardial perfusion indexes for each patient. In addition, our study relies on a feasible and reproducible technique, applanation tonometry, which might be easily performed on patients with HF in several geriatric settings, suggesting that pulse wave analysis may be included in the comprehensive assessment of HF elderly patients, as a short-term outcome and early rehospitalization predictor.

## Conclusion

In conclusion, medical therapy seems to improve arterial stiffness and subendocardial perfusion in geriatric patients hospitalized with heart failure. Noteworthy, PWVcf is a valid predictor of 30-day readmission, and owing to its feasibility and reproducibility in clinical practice, it may provide an instrument to detect patients with HF at high risk of rehospitalization, in order to plan a tailored management and follow-up, in the early phase after hospital discharge.

## Data Availability Statement

The raw data supporting the conclusions of this article will be made available by the authors, without undue reservation.

## Ethics Statement

The studies involving human participants were reviewed and approved by the Ethical Committee University of Verona. The patients/participants provided their written informed consent to participate in this study.

## Author Contributions

FF contributed to conceptualization, methodology, statistical analyses, and writing—original draft. AG contributed to validation, data curation, and writing—original draft. AF and SU contributed to data collection. EZ, GM, and AR contributed to methodology and editing. MZ contributed to conceptualization, methodology, writing—review and editing, and supervision. All authors contributed to the article and approved the submitted version.

## Conflict of Interest

The authors declare that the research was conducted in the absence of any commercial or financial relationships that could be construed as a potential conflict of interest.

## Publisher’s Note

All claims expressed in this article are solely those of the authors and do not necessarily represent those of their affiliated organizations, or those of the publisher, the editors and the reviewers. Any product that may be evaluated in this article, or claim that may be made by its manufacturer, is not guaranteed or endorsed by the publisher.
